# Assessing Choroidal Nevi, Melanomas and Indeterminate Melanocytic Lesions Using Multimodal Imaging—A Retrospective Chart Review

**DOI:** 10.3390/curroncol29020087

**Published:** 2022-02-11

**Authors:** Fredy Geiger, Sadiq Said, Anahita Bajka, Mario Damiano Toro, Maximilian Robert Justus Wiest, Marc Stahel, Daniel Barthelmes, Sandrine Anne Zweifel

**Affiliations:** 1 Department of Ophthalmology, University Hospital of Zurich, 8091 Zurich, Switzerland; fredy.geiger@usz.ch (F.G.); sadiq.said@usz.ch (S.S.); anahita.bajka@usz.ch (A.B.); maximilian.wiest@usz.ch (M.R.J.W.); marc.stahel@usz.ch (M.S.); daniel.barthelmes@usz.ch (D.B.); 2 University of Zurich, 8006 Zurich, Switzerland; 3 Chair and Department of General and Pediatric Ophthalmology, Medical University of Lublin, 20079 Lublin, Poland; toro.mario@email.it; 4 Eye Clinic, Public Health Department, University of Naples Federico II, 80133 Naples, Italy

**Keywords:** multimodal imaging, optical coherence tomography, choroidal tumors, melanoma, choroidal nevus, ophthalmic oncology

## Abstract

Using multimodal imaging, the literature proposed the following risk factors for choroidal nevus growth into melanoma: increased tumor thickness, subretinal fluid, decreased visual acuity, presence of orange pigment, ultrasound acoustic hollowness, and increased tumor diameter. This study investigated the presence of the mentioned risk factors in choroidal nevi, choroidal melanomas, and indeterminate choroidal melanocytic lesions. This retrospective, single-center chart review assessed choroidal melanocytic tumors with multimodal imaging. We defined our primary outcome as the cumulative presence of mentioned risk factors. Further, we evaluated various optical coherence tomography (OCT), ultrasound, and autofluorescence findings. We analyzed 51 tumors from 49 patients during the period from April 2008 to June 2021. The median (IQR) age was 64.0 (56.0 to 70.5) years, with 23 of 49 (46.9%) patients being female. The follow-up time for all tumors was median (IQR) 25.0 (12.0 to 39.0) months. The choroidal nevi had a median (range) risk score of 0.0 (0.0 to 3.0), and the choroidal melanoma of 5.0 (3.0 to 6.0), with statistically significant different ratings (*p* < 0.001). Multimodal imaging creates a score that may help to distinguish choroidal nevi from choroidal melanomas objectively.

## 1. Introduction

Clinicians may broadly classify choroidal melanocytic tumors as benign or malignant lesions, with choroidal nevi comprising the benign counterpart to melanoma. However, both histological evidence [1,2] and documented clinical cases [3,4] indicate that most intraocular melanomas may evolve from such benign uveal lesions. Since biopsy is not favorable for conclusive diagnosis in these tumors, a thorough clinical examination remains of utmost importance. Current expert recommendations for specifying suspicious choroidal nevi include both ophthalmoscopy and multimodal imaging. Fundus photography, wide-field fundus imaging (e.g., Optos) [5], optical coherence tomography (OCT), especially using enhanced depth imaging (EDI) features [6,7,8], (wide-field) fundus autofluorescence [9], and ultrasonography [10] are successfully used to document and diagnose choroidal tumors, to reveal growth and progression in monitoring visits, and hence detect risk factors for transformation. Even with all listed diagnostic tools, certain tumors cannot be accurately classified as choroidal nevus or choroidal melanoma. In these cases, the diagnosis of “indeterminate choroidal melanocytic lesion” is used to designate such tumors [11,12]. Due to the mentioned diagnostic uncertainty, these lesions require particularly careful monitoring.

The ophthalmology department at the University Hospital Zurich acts as a referral center for various intraocular tumors in Switzerland. For choroidal melanocytic lesions, there is a wide variation in diagnostic expertise among referring ophthalmologists. However, the early detection of malignant lesions and the initiation of specific treatment are directly associated with better ocular outcomes and patient survival [13]. In a study published in late 2019, Shields et al. investigated with multimodal imaging the risk factors for transformation of choroidal nevus into melanoma [14]. Using multivariate analyses, they found the following six risk factors for tumor transformation, which are mnemonically referred to as “To Find Small Ocular Melanoma Doing Imaging” (TFSOM-DIM) and denote tumor thickness >2 mm in ultrasound imaging, subretinal fluid detected with OCT, visual acuity of 20/50 or worse according to Snellen, orange pigment detected with autofluorescence, melanoma acoustic hollowness measured with ultrasound, and tumor diameter >5 mm determined with fundus photography [14].

However, the current literature on multimodal imaging for uveal melanocytic lesions is scarce. The primary objective of this study is to assess our clinically made diagnoses based on the above-mentioned six risk factors by investigating our local database of choroidal tumors. We expect to find more risk factors present in choroidal melanomas compared to choroidal nevi or indeterminate choroidal lesions. The results of this study may support and add to the current understanding of risk factors associated with malignancy of choroidal melanocytic lesions.

## 2. Materials and Methods

This is an investigator-initiated, retrospective, single-center study conducted at the University Hospital Zurich in Switzerland. We identified patients clinically diagnosed with choroidal melanoma, choroidal nevus, or indeterminate choroidal melanocytic lesion in our clinic between April 2008 and June 2021. The leading ethics committee in Zurich approved our study according to the human research act (BASEC-No. 2019-02043). All patients agreed in writing to the further use of their clinical data for research purposes. We handled all data according to Good Clinical Practice guidelines.

### 2.1. Data Collection

**Figure 1:**
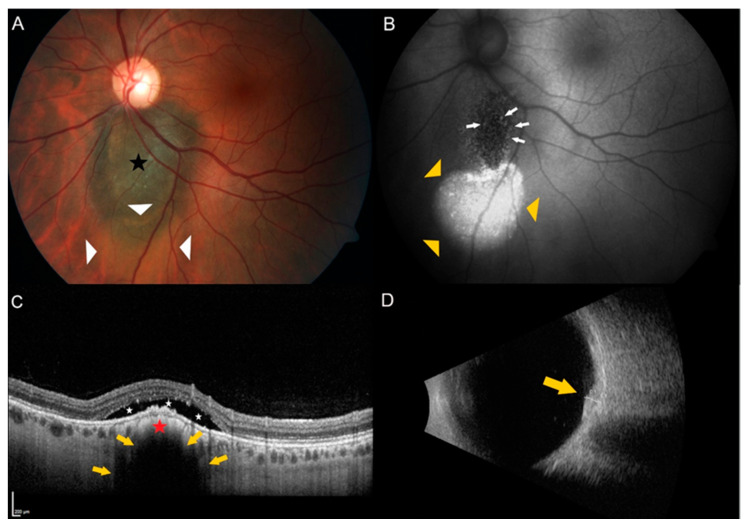
Example of a juxtapapillary indeterminate choroidal melanocytic lesion showing two risk factors. (**A**) Color fundus photography of the left eye of a 35-year old female patient showing a pigmented choroidal lesion (black star) adjacent to the optic disc. Please note the presence of subretinal fluid over and inferior to the lesion (white triangles). (**B**) Autofluorescence imaging highlights the subretinal fluid (yellow triangles) and shows the presence of orange pigment (white arrows) over the lesion. (**C**) Enhanced depth spectral-domain optical coherence tomography (EDI-OCT) of the dome-shaped tumor (red star) shows choriocapillaris compression and choroidal shadowing (yellow arrows) as well as subretinal fluid (white stars) over the tumor. (**D**) B-scan ultrasonography shows a dense, dome-shaped lesion (yellow arrow) measuring 1.5 mm in thickness.

We reviewed our archived consultation reports of patients aged 18 and older with the above-mentioned clinical diagnoses using the keywords choroid, melanoma, and choroidal tumor. The clinical findings were obtained using fundoscopy and multimodal imaging that included fundus photography using the ZEISS camera FF450 version VISUPAC 4.5.2 (Carl Zeiss AG, Oberkochen, Germany), wide-field fundus imaging with Optos 200Tx (Optos, Inc. Marlborough, MA, USA), spectral-domain OCT and autofluorescence both using Heidelberg Spectralis OCT Spectralis version 1.9.10.0 (Heidelberg Engineering GmbH, Heidelberg, Germany), and ultrasound using the Aviso S (Quantel Medical by Lumibird GmbH, Cournon d’Auvergne, France). Study author M.S. is the expert in intraocular tumors at our tertiary care referral hospital and diagnoses all suspicious choroidal lesions. To assess the clinical diagnoses of choroidal melanocytic lesions in our database, we employed the risk factors proposed by Shields et al. in 2019 [14]. We investigated the individual risk factors for each of the 51 tumors. Figure 1 shows an example tumor of an indeterminate choroidal melanocytic lesion with two risk factors present. All patients without or with poor OCT scans of the lesion and patients who were already receiving tumor therapy at the time of the first OCT were excluded.

### 2.2. Baseline and Follow Up Measures

We set the date of the first OCT scans of the tumor as the baseline examination. Reviewing the patients’ medical history, we included their sex and age at that time as demographic data and extracted the side and the baseline diagnosis of the affected eye with its best-corrected visual acuity (VA) using the Snellen method. We defined the last measurement as the follow-up examination, extracting the follow-up time and the corrected final diagnosis.

For our primary outcome, we generated categorical variables following the proposed risk factors by Shields et al. [14]. We considered lesion thickness binary with 2 mm as the cutoff, subretinal fluid binary as present or absent, Snellen visual acuity binary with 20/50 as the cutoff, orange pigment, and tumor acoustic hollowness as present or absent (if no ultrasound image of the lesion was found, hollowness was considered to be absent), and tumor diameter binary with 5 mm as the cutoff value. Thus, a score between 0 and 6 points is possible, with the highest score reflecting all risk factors present.

Further, we included several different secondary measures. We investigated the presence of orange pigment and “retinal trough” using fundus autofluorescence imaging. The term “retinal trough” describes the appearance of a defined zone of RPE atrophy extending outwards from the nevus margin [15,16]. Using ultrasound, we examined tumor thickness, its shape, and echogenicity. Using OCT, we investigated the following parameters: subretinal fluid overlying the tumor, subretinal fluid <3 mm from the tumor margin, subfoveal fluid, retinal invasion, retinal edema over the tumor, drusen above the tumor, shaggy photoreceptors overlying the tumor, loss of the ellipsoid zone, irregularity of the ellipsoid zone, retinal pigment epithelium (RPE) atrophy, RPE hyperplasia or RPE fibrous metaplasia over the tumor, RPE detachment or choroidal neovascularization over the tumor, surface tumor configuration (such as dome-shaped, “lumpy bumpy”, excavated, or flat), compression of the choriocapillaris and the tumor margin closer than 3 mm to the optic disc. If tumors appeared thinner than 3 mm in US, study authors F.G. and A.B. also measured their thickness with OCT. Whenever possible, we used the ultrasound thickness measurements for further statistical analyses, such as calculating the individual risk score. However, we considered the OCT thickness measurement if we did not find an ultrasound image of the tumor.

Regarding the largest basal tumor diameter, the same study authors mostly assessed fundus photography. If no photography was present, we used wide-field fundus imaging (Optos) to measure mentioned diameters. We defined the largest basal diameter in the baseline assessment as the mean of both study authors’ measurements.

Finally, we examined the electronic charts for any tumor-targeted therapeutic actions and tumor-related complications such as secondary choroidal neovascularization (CNV), accompanying retinal detachment, toxic tumor syndrome, or any form of metastases. We transferred all data to a spreadsheet using Excel (Microsoft Excel 2016, Microsoft Corporation, Redmond, Washington, DC, USA).

### 2.3. Statistical Analyses

To give a descriptive overview, we presented means with standard deviation and medians with interquartile ranges (IQR) or minimum to maximum values for continuous data, and numbers and percentages for categorical data. Regarding the calculated risk scores, we applied the Kruskal Wallis test to analyze these categorical data. Please note that comparisons are exploratory. Therefore *p*-values were not adjusted for multiple comparisons. For all analyses, we considered *p* < 0.05 to indicate statistical significance.

## 3. Results

**Figure 2:**
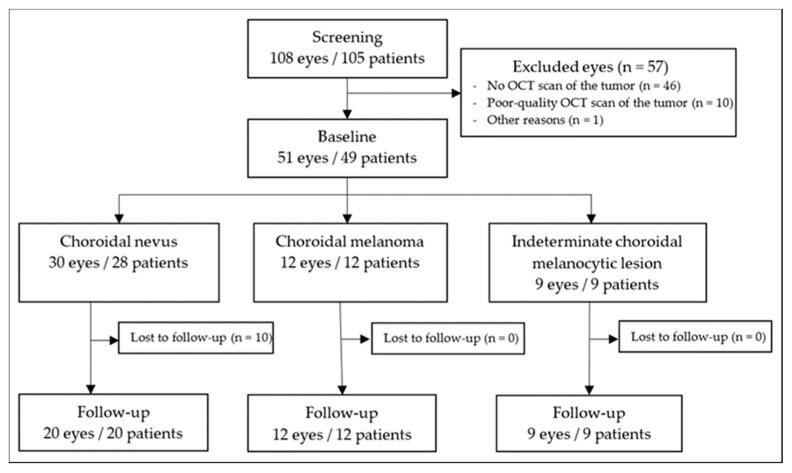
Flowchart illustrating study inclusion and exclusion. OCT = optical coherence tomography.

We examined our database between April 2008 and June 2021 concerning our inclusion and exclusion criteria. In total, we analyzed 51 choroidal melanocytic tumors from 49 patients. Reviewing the diagnoses made in our archived consultation reports, we found 58.8% (30 of 51) choroidal nevi, 23.5% (12 of 51) choroidal melanomas, and 17.6% (9 of 51) indeterminate choroidal melanocytic lesions. Of those 51 tumors, 10 choroidal nevi were lost to follow-up. Figure 2 illustrates the data collection as a flowchart. The follow-up time for all tumors was median (IQR) 25.0 (12.0 to 39.0) months. During the entire observation period, no choroidal nevus and no indeterminate choroidal melanocytic lesion transformed into a choroidal melanoma. The overall median (IQR) age was 64.0 (56.0 to 70.5) years, with 23 of 49 (46.9%) patients being female. Table 1 displays the study and patient characteristics in detail.

**Figure 3:**
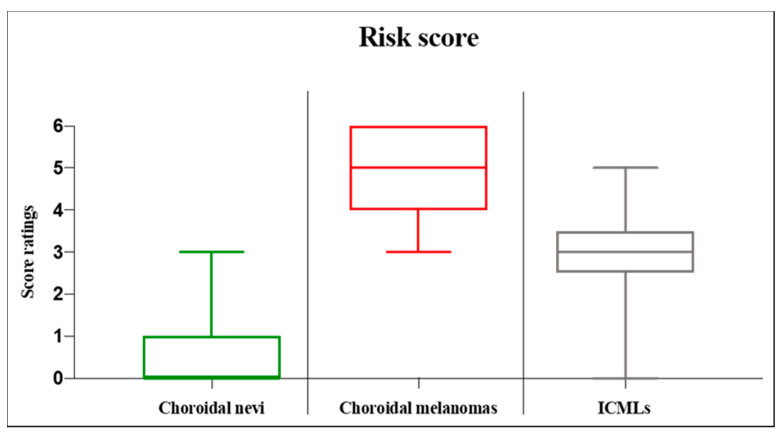
Boxplots displaying the analysis of the risk score for choroidal melanocytic lesions. The score consists of the following risk factors proposed by Shields et al. regarding choroidal nevus transformation to melanoma [14]: lesion thickness, subretinal fluid, Snellen visual acuity, orange pigment, ultrasound acoustic hollowness, and tumor diameter. A cumulative score between 0 and 6 points is possible, with the highest score reflecting all risk factors present. The box displays the first and third quartiles, and the line within the median value. The whiskers represent the minimum and maximum value. ICML = indeterminate choroidal melanocytic lesion.

The risk factors were assessed using the Kruskal Wallis test. In 23 cases of choroidal nevi with missing ultrasound imaging, we used OCT thickness measurements for the primary outcome. All OCT thickness measurements mentioned were less than 1 mm, with the highest value being 557μm. We report the following results as median (minimum to maximum). The overall score of the tumors was 1.0 (0.0 to 6.0). The choroidal nevi had a score of 0.0 (0.0 to 3.0), and the choroidal melanoma of 5.0 (3.0 to 6.0), with a statistically significant different rating (*p* < 0.001). With median values of 3.0 (0.0 to 5.0) for indeterminate choroidal melanocytic lesions, we found a statistically significant difference when comparing to both other diagnoses (*p* < 0.001 and *p* = 0.002, respectively in order). Figure 3 displays the risk score analysis as boxplots.

Regarding choroidal nevi in OCT, most tumors presented as flat (23 of 30, 76.7%). Subretinal fluid (6 of 30, 20.0%) and retinal edema (1 of 30, 3.3%) were features rarely found, and there were no shaggy photoreceptors detected over mentioned lesions. Regarding choroidal melanomas, all presented as dome-shaped and had overlying subretinal fluid in OCT imaging (12 of 12, 100.0%), with 6 out of 12 (50%) having shaggy photoreceptors above the lesions. Further, we found retinal edema over the tumor in 7 out of 12 (58.3%) eyes. Assessing the indeterminate choroidal melanocytic lesions, seven out of nine (77.8%) presented as dome-shaped. Most showed subretinal fluid (seven of nine, 77.8%) and retinal edema (five of nine, 55.6%). We detected shaggy photoreceptors over mentioned lesions in two out of nine (22.2%) cases. Regarding autofluorescence imaging, we found the occurrence of overlying orange lipofuscin pigment in 1 out of 30 (3.3%) choroidal nevi, in 10 out of 12 (83.3%) choroidal melanomas, and in seven out of nine (77.8%) indeterminate choroidal melanocytic lesions. We found no RPE through in any of our assessed patients. Table 2 provides a detailed overview of the descriptive analyses of assessed OCT features.

Table 3 lists the detailed descriptive analyses regarding the ultrasound imaging features and the largest basal diameter. Please note that the thickness values provided only represent the ultrasound measurements and not the OCT thickness readings. We found inconsistent reporting with 70.0% (21 of 30) missing data in the choroidal nevi group. However, both other diagnostic groups were fully reported. The choroidal melanomas were described as hollow in most cases (10 of 12, 83.3%) and the indeterminate lesions as dense (6 of 9, 66.7%). Regarding the median (IQR) ultrasound tumor thickness measurements in millimeters, choroidal melanomas were thicker 2.50 (2.28 to 4.16) than the indeterminate melanocytic lesions 1.52 (1.46 to 1.69) and the choroidal nevi 1.14 (0.97 to 1.45).

In Table 4, we provide the tumor-related complications. Assessing the therapeutic measures, all patients diagnosed with choroidal melanoma received proton beam radiotherapy (12 out of 12, 100.0%). Additionally, one case received laser photocoagulation (1 of 12, 8.3%), one case photodynamic therapy (1 of 12, 8.3%), and one a systemic carboplatin-based chemotherapy due to liver metastases (1 of 12, 8.3%).

Regarding the injection of anti-vascular endothelial growth factors (anti-VEGF), one patient with a choroidal nevus (1 of 30, 3.3%), ten patients with choroidal melanomas (10 of 12, 83.3%), and one with an indeterminate choroidal melanocytic lesion (1 of 9, 11.1%) received treatment.

## 4. Discussion

This study retrospectively investigated our local database of choroidal melanocytic lesions. Considering our in- and exclusion criteria, we assessed a total of 51 choroidal tumors. The melanomas showed significantly more risk factors for malignancy than choroidal nevi. Analyzing our database descriptively, the lesions seemed to differ regarding specific OCT findings. With advances and the increasing availability of multimodal fundus imaging [17] but varying diagnostic competence concerning intraocular tumors, reliable diagnostic criteria will be of enduring importance [18].

Although Shields et al. suggested using mentioned imaging and clinical features as predictive factors to evaluate the risk for choroidal nevi growth into melanomas [14], this study assessed the cumulative occurrence of mentioned factors regarding each pathology. In patients diagnosed with choroidal melanoma, we found significantly higher risk scores than in patients with choroidal nevus. Note that we used OCT thickness measurements in some choroidal nevi cases to calculate the binary thickness score. However, we believe that this change in measurement method did not affect our primary outcome as all mentioned nevi were far thinner than 1 mm. The literature showed that OCT thickness measurements might be appropriate for such thin choroidal tumors due to several advantages, such as less misjudgment of thickness compared to ultrasound [19]. It seems that the risk score compares well with expert diagnosis and may be applied not only to assess the risk of transformation, but also to distinguish between the different diagnoses at a given point in time. However, this tumor scoring system requires several imaging devices, whereby ultrasound in particular is limited in availability [20]. Al Harby et al. reported in 2021, using a different scoring system, that tumor thickness measurements with ultrasonography influenced tumor categorization in nevi or melanomas in only 6 of 222 (2.7%) cases [21]. Hence, future studies with a larger amount of data are necessary to examine whether ultrasound measurements can be omitted to assess malignancy. This reduction in required diagnostic technologies may ease tumor differentiation, especially in private ophthalmologic clinics.

In this study cohort, the median age for patients with choroidal nevi and indeterminate choroidal melanocytic lesions was 65 years and for choroidal melanomas, 62 years. Considering choroidal melanomas in Caucasian people, other epidemiological studies report a similar median age around 60 years [22,23,24]. Choroidal melanomas seem to occur more frequently during the transition between middle and older adulthood. Concerning choroidal nevi, determining an average age of diagnosis is difficult as these tumors seem to be found in quasi every age group, whereas they are rarely seen in young patients under 20 years of age [15]. In one of the only population-based studies in Caucasians, the highest prevalence of choroidal nevi was found between 50 and 60 years [25]. Comparing our study with larger trials investigating only choroidal nevi, the median age is similarly at approximately 60 years [14,15,26]. Nevertheless, the studies mentioned above report a broad age distribution. The choroidal tumor population of our study cohort appears to be a representative epidemiologic sample for the Caucasian population.

Assessing our database on OCT imaging, we found shaggy photoreceptors in half of the choroidal melanoma cases but none in patients with choroidal nevi. The indeterminate lesions seem to lie in between, with 22.2% of the eyes showing the mentioned feature. This OCT term has been used to describe irregularly shaped, prolonged, and presumed swollen photoreceptors from subretinal fluid [19]. We found similar results regarding their occurrence compared to the literature [19]. However, Shields et al. also reported detecting shaggy photoreceptors in 73 of 232 (32%) cases of choroidal nevi that displayed subretinal fluid [27]. Further, this OCT finding has been previously described in other diseases such as choroidal metastases [28], central serous chorioretinopathy [29], and in patients with sympathetic ophthalmia [30]. Shaggy photoreceptors are not unique to choroidal melanomas. Reporting other OCT features examined in this study, we found subretinal fluid, retinal edema over the lesion, a change in the ellipsoid zone over the tumor, and RPE changes over the lesion like RPE atrophy or RPE hyperplasia more frequently in patients with choroidal melanomas than with choroidal nevi. These findings align with the current literature [14,19,31] and thus strengthen the understanding of OCT-guided discrimination between the two pathologies. As for the appearance of indeterminate choroidal melanocytic lesions on OCT and autofluorescence imaging, some studies show that they resemble choroidal melanomas [12,32], which we also found in our investigation. Hence, patients with such indeterminate choroidal melanocytic lesions should be further examined and closely monitored by preferably an ocular oncologist [33].

The strengths of this study include the use of multimodal imaging and the broad evaluation of OCT features. However, there are several limitations. First, retrospective design is inherently prone to selection bias as well as missing data. In our study, tumor thickness measurements by ultrasound were often omitted for choroidal nevi because the lesions appeared clinically flat and benign. These missing data may have influenced the retrospective analyses. Second, all diagnoses were made clinically. Histological evaluation may invalidate the diagnoses made and, in indeterminate choroidal melanocytic lesions, provide additional information such as genetic prognostic markers that may aid treatment decision-making [34]. Although biopsy is becoming more common, it is usually limited to lesions thicker than 1.5 mm and an accessible location without risk of vision loss. Third, the overall sample size is comparably small. Results may change if more patients were included. However, this study enrolled a reasonably sized number of cases for a country with a small population like Switzerland. Finally, we assessed patients in a tertiary care hospital in central Europe with mainly Caucasian patients. Tumor prevalence and patient demographics may alter elsewhere in the world.

## 5. Conclusions

Multimodal imaging with fundus photography, OCT, autofluorescence, and ultrasonography, along with visual acuity assessment, creates a score that may help to distinguish choroidal nevi from melanomas objectively. The score is based on the following factors: tumor thickness > 2 mm, subretinal fluid, visual acuity of 20/50 or worse, detection of orange pigment, ultrasound acoustic hollowness, and tumor diameter > 5 mm. Patients with an increased cumulative score should especially be referred to ocular oncologists for further tumor workup. Future studies should re-evaluate the above risk score using a larger study population.

## Figures and Tables

**Table 1:** Patient and demographic characteristics at the baseline examination. We divided all clinical diagnoses into the following three groups: choroidal nevus, choroidal melanoma, and indeterminate choroidal melanocytic lesion. We report visual acuity using the Snellen method. Data presented as number (%) or median (IQR [range]). ICML = indeterminate choroidal melanocytic lesion.

	Choroidal Nevus	Choroidal Melanoma	ICML	Total
Age; years	65 (55.0–71.0 [27–87])	62 (57.0–65.5 [30–77])	65 (62.0–66.0 [35–81])	64 (56.0–70.5 [27–87])
Sex; female	16 of 28(57.1%)	5 of 12 (41.7%)	2 of 9 (22.2%)	23 of 49 (46.9%)
Affected eye; right	14 of 30 (46.7%)	7 of 12 (58.3%)	2 of 9 (22.2%)	23 of 51 (45.2%)
Baseline diagnosis	30 of 51(58.8%)	12 of 51(23.5%)	9 of 51(17.6%)	51
Follow-up diagnosis	20 of 41 (48.8%)	12 of 41 (29.3%)	9 of 41(22.0%)	41
Follow-up time; months	15.5 (10.0–39.0 [0.5–79.0])	30.0 (20.8–44.0 [2.0–134.0])	15.0 (14.0–37.0 [11.0–75.0])	25.0 (12.0–39.0 [0.5–134.0])
Baseline visual acuity	20/20 (20/20–20/25 [20/16–20/40])	20/32 (20/25–20/63 [20/20–20/400])	20/20 (20/20–20/25 [20/16–20/2000])	20/20 (20/20–20/25 [20/16–20/2000])
Follow-up visual acuity	20/22 (20/20–20/32 [20/20–20/50])	20/56 (20/30–20/400 [20/20-20/4000])	20/20 (20/20–20/32 [20/20–20/2000])	20/25 (20/20–20/36 [20/20–20/4000])

**Table 2:** Spectral-domain optical coherence tomography (SD-OCT) features. Data presented as number (%). We assessed 15 OCT features. *N* = 51 total tumors, *N*1 = 30 choroidal nevi, *N*2 = 12 choroidal melanomas, *N*3 = nine indeterminate choroidal melanocytic lesions. ICML = indeterminate choroidal melanocytic lesion; mm = millimeter; RPE = retinal pigment epithelium; CNV = choroidal neovascularization.

	Choroidal Nevus, *N*1	Choroidal Melanoma, *N*2	ICML, *N*3
**Subretinal fluid** Overlying tumor Within 3 mm from tumor margin Subfoveal	3 (10.0%) 3 (10.0%) 2 (6.7%)	12 (100.0%) 0 (0.0%) 8 (66.7%)	7 (77.8%) 0 (0.0%) 2 (22.2%)
Retinal invasion	1 (3.3%)	0 (0.0%)	0 (0.0%)
Retinal edema over tumor	1 (3.3%)	7 (58.3%)	5 (55.6%)
Drusen above tumor	14 (46.7%)	1 (8.3%)	3 (33.3%)
Shaggy photoreceptors overlying tumor	0 (0.0%)	6 (50.0%)	2 (22.2%)
Loss of ellipsoid zone	6 (20.0%)	9 (75.0%)	8 (88.9%)
Irregularity of ellipsoid zone	8 (26.7%)	12 (100.0%)	7 (77.8%)
RPE atrophy	6 (20.0%)	11 (91.7%)	8 (88.9%)
RPE hyperplasia	4 (13.3%)	8 (66.7%)	5 (55.6%)
RPE fibrous metaplasia	1 (3.3%)	2 (16.7%)	0 (0.0%)
RPE detachment	5 (16.7%)	8 (66.7%)	2 (22.2%)
CNV	3 (10.0%)	1 (8.3%)	1 (11.1%)
Choriocapillaris compression	26 (86.7%)	12 (100.0%)	9 (100.0%)
**Surface configuration** Dome-shaped Lumpy bumpy Excavated Flat	6 (20.0%) 1 (3.3%) 0 (0.0%) 23 (76.7%)	12 (100.0%) 0 (0.0%) 0 (0.0%) 0 (0.0%)	7 (77.8%) 0 (0.0%) 0 (0.0%) 2 (22.2%)
Tumor margin <3 mm to the optic disc	7 (27.5%)	4 (33.3%)	3 (33.3%)

**Table 3:** Ultrasound and fundus imaging features. We assessed the tumor shape, its echogenicity and thickness using ultrasound, and the largest basal diameter using fundus photography. Data presented as number (%) or median (IQR [range]). ICML = indeterminate choroidal melanocytic lesion.

	Choroidal Nevus		Choroidal Melanoma	ICML
**Shape** Flat configuration Dome configuration Not available	4 of 9 (44.4%) 5 of 9 (55.6%) 21 of 30 (70.0%)		0 of 12 (0.0%) 12 of 12 (100.0%) 0 of 12 (0.0%)	3 of 9 (33.3%) 6 of 9 (66.7%) 0 of 9 (0.0%)
**Echogenicity** Hollow Dense Not available	2 of 9 (22.2%) 7 of 9 (77.8%) 21 of 30 (70.0%)		10 of 12 (83.3%) 2 of 12 (16.7%) 0 of 12 (0.0%)	3 of 9 (33.3%) 6 of 9 (66.7%) 0 of 9 (0.0%)
Thickness; millimeter	1.1 (1.0–1.5 [0.8–1.9])		2.5 (2.3–4.2 [1.4–6.0])	1.5 (1.5–1.7 [1.1– 2.1])
Largest basal diameter; millimeters	3.5 (2.2–4.6 [1.0–8.0])		10.4 (5.2–12.7 [3.9–17.6])	6.1 (5.5–8.3 [3.3–11.4])

**Table 4:** Tumor-related complications. Data presented as number (%). *N* = 51 total tumors, *N*1 = 30 choroidal nevi, *N*2 = 12 choroidal melanomas, *N*3 = nine indeterminate choroidal melanocytic lesions. ICML = indeterminate choroidal melanocytic lesion; CNV = choroidal neovascularization.

	Choroidal Nevus, *N*1	Choroidal Melanoma, *N*2	ICML, *N*3	Total, *N*
Secondary CNV	3 (10.0%)	1 (8.3%)	1 (11.1%)	5 (9.8%)
Retinal detachment	0 (0.0%)	5 (41.7%)	1 (11.1%)	6 (11.8%)
Toxic tumor syndrome	0 (0.0%)	1 (8.3%)	0 (0.0%)	1 (2.0%)
Metastasis	0 (0.0%)	1 (8.3%)	0 (0.0%)	1 (2.0%)

## Data Availability

Data will be made available upon request from the corresponding author.
